# Engineering PVA-CNF-MOF Composite Films for Active Packaging: Enhancing Mechanical Strength, Barrier Performance, and Stability for Fresh Produce Preservation

**DOI:** 10.3390/molecules30193971

**Published:** 2025-10-03

**Authors:** Sergio Carrasco, Juan Amaro-Gahete, Eduardo Espinosa, Almudena Benítez, Francisco J. Romero-Salguero, Alejandro Rodríguez

**Affiliations:** 1 BioPrEn Group (RNM940), Chemical Engineering Department, Instituto Químico para la Energía y el Medioambiente (IQUEMA), Faculty of Science, University of Córdoba, 14014 Córdoba, Spain; q62carcs@uco.es (S.C.); a02esvie@uco.es (E.E.); q42ropaa@uco.es (A.R.); 2 Materiales Polifuncionales Basados en Carbono (UGR-Carbon), Department of Inorganic Chemistry, Unidad de Excelencia Química Aplicada a Biomedicina y Medioambiente, University of Granada (UEQ-UGR), 18071 Granada, Spain; 3 Deparment of Organic Chemistry, Instituto Químico para la Energía y el Medioambiente (IQUEMA), Faculty of Science, University of Córdoba, Campus de Rabanales, Ed. Marie Curie, 14071 Córdoba, Spain; qo2rosaf@uco.es; 4 Departament of Inorganic Chemistry and Chemical Engineering, Instituto Químico para la Energía y el Medioambiente (IQUEMA), Faculty of Science, University of Córdoba, 14071 Córdoba, Spain; q62betoa@uco.es

**Keywords:** metal-organic frameworks, cellulose nanofibers, PVA, PVA-CNF-MOF film, active packaging

## Abstract

Food waste is a global challenge, with nearly 40% of food discarded annually, leading to economic losses, food insecurity, and environmental harm. Major factors driving spoilage include microbial contamination, enzymatic activity, oxidation, and excessive ethylene production. Active packaging offers a promising solution by extending shelf life through the selective absorption or release of specific substances. In this study, polyvinyl alcohol (PVA) films incorporating metal-organic frameworks (MOFs) were prepared via solvent casting to enhance their mechanical and barrier properties. Five MOFs (HKUST-1, MIL-88A, BASF-A520, UiO-66, and MOF-801) were embedded in the PVA matrix and analyzed for their physical, mechanical, and optical characteristics. The incorporation of TEMPO-oxidized cellulose nanofibers (CNF) improved MOF dispersion, significantly strengthening film performance. Among the formulations, PVA-CNF-MOF-801 exhibited the best performance, with a 130% increase in tensile strength, a 50% reduction in water vapor permeability, and a 168% improvement in UV protection compared with neat PVA films. Ethylene adsorption tests with climacteric fruits confirmed that CNF-containing films retained ethylene more effectively than those without CNFs, although the differences among the MOFs were minimal. These results highlight the potential of PVA-CNF-MOF composite films as sustainable active packaging materials, providing an effective strategy to reduce food waste and its environmental impact.

## 1. Introduction

The global food supply chain faces considerable hurdles due to population growth, which is projected to reach 9.8 billion inhabitants by 2050 [1], compounded by intensifying environmental pressures driven by climate change, socio-economic disruptions in the post-COVID-19 era that have exposed critical vulnerabilities in global supply chains, and geopolitical instabilities such as the Russia-Ukraine conflict, which has markedly affected the availability and cost of key agricultural commodities [2,3,4]. Several studies suggest that the increase in population will require a greater food supply, which is expected to increase by 35% to 56% between 2010 and 2050 [5], leading to a rise in food waste [6]. According to the latest available data, nearly 40% of the food intended for human consumption (1.3 billion tons of edible food) ends up as waste [7]. This substantial waste, known as “food loss and waste” (FLW), represents a hazard to the economy, environment, and food security [8]. FLW, estimated to be worth 1 trillion US dollars [9], is very harmful to the environment due to decomposition in landfills, where part of it breaks down into greenhouse gases and methane, with a global warming potential 28–36 times higher than carbon dioxide [10]. In fact, FLW contributes to 8–10% of greenhouse gas emissions, with an estimated carbon footprint of 3.3 billion tons of CO_2_ [11]. Consequently, the United Nations (UN) has implemented measures to reduce FLW as part of the Sustainable Development Goals (SDG 12) for 2030 [12]. Solutions to this problem include reducing the amount of waste generated, optimizing the food chain, increasing consumer awareness [13], and improving packaging systems that increase the shelf-life of food [6].

The longevity of food products can be influenced by numerous factors, including microbial contamination, enzymatic degradation, oxidation, or the presence of excessive ethylene [14]. To mitigate these challenges, a range of preservation methods have been devised to extend shelf life, such as coating preservation, vacuum preservation, and active packaging [15]. Among these options, active packaging is emerging as a newer approach that is gaining prominence over traditional packaging systems due to the ability of this type of packaging to control the amount of moisture, oxygen, or ethylene in contact with food. Additionally, active packaging can desorb substances with antimicrobial and antioxidant properties, thus enhancing food preservation. Its versatility and flexibility, driven by its ability to be customized for different applications, make it a favorable field for future research and innovation [15].

In this context, the utilization of metal-organic frameworks (MOFs) is gaining significance in active packaging materials. MOFs represent a class of hybrid porous materials composed of metal ions or clusters coordinated to organic ligands. These materials stand out for their high specific surface area and porosity, presenting a high variation in components in their structure, thus facilitating interaction with multiple materials [16]. One of the main problems faced by MOFs is that they are mainly presented in powder form, which hinders their processing and therefore their direct application or use on an industrial scale [17]. This can be solved by mixing MOFs with other types of materials, such as polymers, forming different types of composites, such as films, membranes, or foams, with the aim of increasing their application and improving the synergistic properties of both components [16,18].

MOFs have garnered significant attention due to their exceptional properties in thin film materials. These hybrid structures incorporate an organic ligand, which imparts functional versatility, and an inorganic substrate, which contributes to mechanical rigidity and stability. Although the integration of inorganic substrates ensures robust mechanical performance and long-term stability, it often involves high costs and intricate fabrication processes [16]. In contrast, polymer membranes, such as polyvinyl alcohol (PVA), offer a more economically viable and industrially feasible alternative. By combining MOFs with PVA, composite films are created that synergistically exploit the adaptable functionality and porosity of MOFs alongside the cost-effectiveness, flexibility, and processability of PVA. This approach not only reduces fabrication complexity but also broadens the scope for scalable and practical applications in various industries. [19]. However, challenges arise during the preparation process, as MOF particles may undergo coverage or aggregation, potentially diminishing their effectiveness. Notwithstanding these challenges, the versatility and potential applications of MOF/PVA composite films, ranging from catalysis to food packaging [20,21], position them as a promising area of research and development in materials science. Addressing issues related to particle coverage and aggregation could further enhance the performance and applicability of these composite materials across various industries.

In this aspect, the incorporation of TEMPO-oxidized cellulose nanofibers (CNFs), produced through a selective oxidation process using the TEMPO (2,2,6,6-tetramethylpiperidine-1-oxyl) catalyst, offers an intriguing solution as these nanofibers exhibit a high content of carboxyl groups and a large specific surface area, enabling improved interactions with the positive charge of metal ions. This strategy would improve the dispersion of MOF particles and ensure a better homogenization in the PVA composite film resulting in a more effective synthesis procedure. Also, CNF adhesion to PVA films has been shown to be efficient since it improves mechanical properties and thermal stability as well as reduces water vapor permeability and water uptake [22,23]. The integration of the mechanical properties of PVA-CNF films with the capacity of MOFs for the adsorption and desorption of bioactive compounds positions these composite materials as highly suitable for diverse applications, including active packaging [24]. Notably, certain MOFs have demonstrated exceptional suitability for ethylene retention [25], a key factor in the preservation of fruits and vegetables, thereby significantly extending their shelf life.

This study focuses on the production of MOF/PVA composite films through a simple solution casting process [26]. Five types of MOFs (MIL-88A, A520-BASF, HKUST-1, MOF-801, and UiO-66) were compared to evaluate their influence on the physicochemical properties of the resulting materials. MOF/PVA films were characterized to determine their physical properties (density, moisture content, water solubility, swelling behavior, and water vapor permeability), mechanical properties (tensile strength and Young’s modulus), and optical properties (transparency and UV-light barrier). Their structural and morphological features were further analyzed using X-ray diffraction (XRD), Fourier transform infrared spectroscopy (FTIR), and scanning electron microscopy (SEM). To improve the interaction between the film components, TEMPO-oxidized cellulose nanofibers, obtained to our knowledge for the first time from cotton linters, were incorporated. In this way, MOF-PVA-CNF films were developed and characterized using a similar methodology. In addition to their role as physical barriers, these composite films were designed to provide active functionalities relevant to food preservation, including enhanced UV shielding, improved barrier properties against water vapor, and ethylene scavenging capacity, thereby contributing to the delayed ripening and reduced spoilage of fresh produce. Both MOF-PVA and MOF-PVA-CNF composite films were subsequently tested on climacteric fruits to assess their ethylene adsorption capacity, demonstrating their potential as sustainable active materials for food packaging.

## 2. Results and Discussion

### 2.1. MOF Characterization

Table 1 summarizes the structural and adsorption properties of the synthesized MOFs, highlighting their potential for ethylene capture based on previous studies [26]. Ethylene adsorption in these materials depends on their specific surface area, pore structure, and active sites. HKUST-1 exhibits the highest ethylene adsorption capacity per gram due to its large surface area. However, when adsorption is normalized per unit area, MIL-88A demonstrates the highest specific ethylene adsorption, surpassing even HKUST-1. This suggests that MIL-88A possesses a high density of active adsorption sites despite its lower total surface area. Ethylene adsorption in MIL-88A is primarily driven by metal-π interactions, in which open metal sites (Lewis acid sites) play a crucial role. A similar mechanism could be expected in other MOFs with accessible metal centers, such as HKUST-1 (Cu-based), BASF-A520 (Al-based), and UiO-66 and MOF-801 (Zr-based). However, their interaction efficiency may vary due to differences in Lewis acidity, pore structure, and metal-ligand coordination environments. While HKUST-1 and BASF-A520 contain transition metals that could contribute to strong ethylene interactions, UiO-66 and MOF-801 feature more stable Zr coordination, which might favor selective adsorption while maintaining good structural integrity.

From a food safety perspective, the biocompatibility and stability of MOFs must be considered. Zirconium-based MOFs (UiO-66 and MOF-801) are generally regarded as highly stable and suitable for applications involving food contact. Aluminum-based MOFs, such as BASF-A520, also exhibit good stability, which can be advantageous for long-term use. HKUST-1, being Cu-based, may require careful consideration regarding potential metal leaching, though its performance in adsorption remains high. Similarly, MIL-88A (Fe-based) presents an interesting balance between adsorption efficiency and biocompatibility, as iron is an essential element in biological systems. Additionally, the choice of organic linkers influences toxicity; thus, selecting chemically stable and non-reactive ligands ensures safe application in active food packaging. Fumaric acid-based linkers, present in MIL-88A, BASF-A520, and MOF-801, are generally considered less toxic, as fumarate is a naturally occurring metabolite involved in biological cycles such as the Krebs cycle in humans [27]. In contrast, aromatic carboxylate ligands, such as benzene-1,3,5-tricarboxylate (HKUST-1) and benzene-1,4-dicarboxylate (UiO-66), may require further evaluation due to potential concerns associated with aromatic compounds [28]. However, the overall toxicity MOFs is influenced not only by its linker but also by its metal center and structural stability, making case-by-case assessments essential for food-related applications.

When considering the integration of MOFs into a PVA matrix with CNF, several factors must be considered, including particle dispersion, interfacial compatibility, and potential hydrogen bonding or electrostatic interactions. MOFs with higher surface areas and more polar functional groups may exhibit better dispersion and interaction with PVA and CNF, leading to enhanced mechanical and barrier properties. In this regard, BASF-A520 [Al(OH)(Fum)·3.5H_2_O], UiO-66 [Zr_6_O_4_(OH)_4_(BDC)_6_], and MOF-801 [Zr_6_O_4_(OH)_4_(Fum)_6_] could be strong candidates due to their structural stability, high surface area, and the presence of hydroxy functional groups coordinated to the metal cluster, which may favor interactions with PVA and CNF via hydrogen bonding. Conversely, MIL-88A [Fe_3_O(Fum)_3_(H_2_O)_2_], which contains fumaric acid linkers in its molecular structure, also exhibits high hydrophilicity, potentially improving dispersion within the polymeric matrix. However, the dynamic structural flexibility (breathing effect) of MIL-88A may influence its performance, depending on the processing conditions. HKUST-1 [Cu_3_(BTC)_2_] despite its high surface area, may require careful consideration due to its potential sensitivity to moisture and its aromatic linker, which could affect its compatibility with the hydrophilic nature of PVA and CNF. Overall, while all MOFs appear suitable for incorporation into the PVA-CNF system, Zr-based MOFs (UiO-66 and MOF-801) and fumaric acid-linked MOFs (MIL-88A and BASF-A520) may provide the best combination of stability, compatibility, and functional properties.

**Figure 1:**
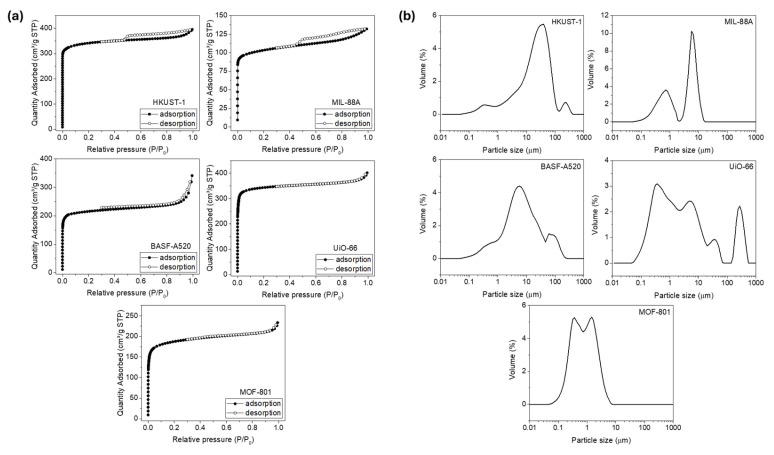
(**a**) N_2_ adsorption-desorption isotherms at −196 °C and (**b**) particle size distribution of MOF samples: HKUST-1, MIL-88A, BASF-A520, UiO-66, and MOF-801.

The N_2_ adsorption-desorption isotherms presented in Figure 1a provided insights into the textural characteristics of the studied MOF samples (Table 2). The isotherms for all samples predominantly exhibited Type I isotherms, which are characteristic of microporous materials [29]. This is evidenced by the steep rise at low relative pressures (P/P_0_), indicating significant micropore filling due to strong adsorbent-adsorbate interactions. However, the isotherms for HKUST-1 (a) and MIL-88A (b) also displayed characteristics of Type IV isotherms, typically associated with mesoporous materials [26,30]. These features were observed in the form of a gradual slope and the presence of hysteresis loops at higher relative pressures, suggesting a combined micro- and mesoporous structure. Specifically, the hysteresis observed in these two samples corresponds to Type IV hysteresis, indicative of capillary condensation occurring in mesopores. This suggested that HKUST-1 and MIL-88A exhibit a hybrid micro- and mesoporous structure. In contrast, the remaining samples lacked significant hysteresis, confirming their predominantly microporous nature [31,32,33]. The textural properties summarized in Table 2 support these observations. Among them, HKUST-1 and UiO-66 achieved the highest Langmuir surface areas (1526 and 1531 m^2^/g, respectively) and micropore volumes (0.584 and 0.585 cm^3^/g), strongly confirming their dominant microporous nature [34,35]. MIL-88A, although having the lowest Langmuir surface area (469 m^2^/g) and total pore volume (0.198 cm^3^/g), displayed an average pore width of 4.9 nm, indicative of some degree of mesoporosity within its structure [26]. In contrast, BASF-A520 presented the largest average pore width (10.6 nm) and a significant total pore volume (0.414 cm^3^/g), consistent with part of its mesoporous framework [31]. Similarly, MOF-801 exhibited a combination of microporosity and mesoporosity, with a total pore volume of 0.333 cm^3^/g and an average pore width of 4.9 nm. The particle size distribution determined by laser diffraction for each MOF is presented in Figure 1b, with the following average particle sizes (D [4.3]) determined for the samples: MIL-88A (4.0 µm), BASF-A520 (17.0 µm), HKUST-1 (31.5 µm), MOF-801 (1.0 µm), and UiO-66 (37.3 µm). These values may be overestimated due to particle aggregation and deviations from sphericity in ethanolic dispersion, as the laser diffraction method assumes an equivalent spherical diameter that may not fully represent the morphology of irregularly shaped or aggregated MOF particles. Such effects contribute to the broader particle size distributions observed in some of the samples.

**Figure 2:**
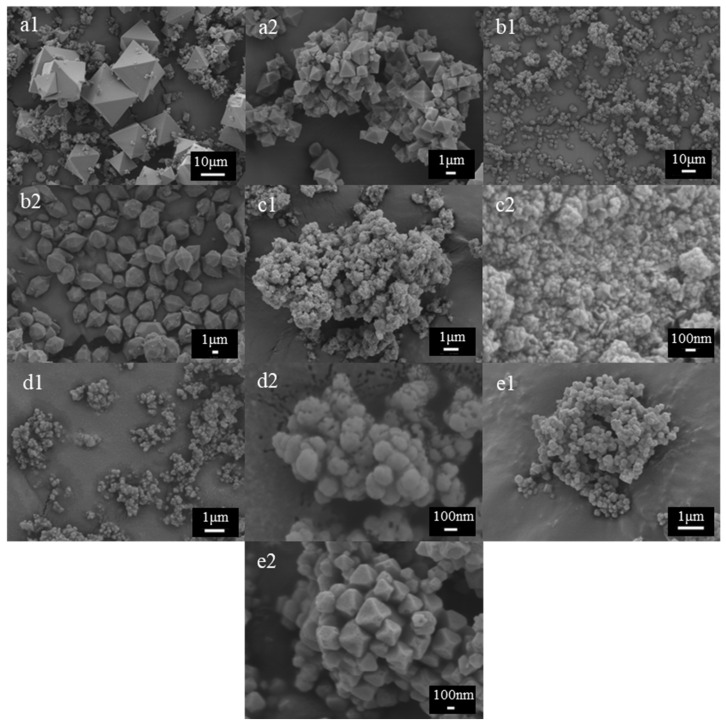
SEM images of MOFs obtained at different magnifications. These correspond to (**a1**,**a2**) HKUST-1, (**b1**,**b2**) MIL-88A, (**c1**,**c2**) BASF-A520, (**d1**,**d2**) UiO-66, and (**e1**,**e2**) MOF-801.

Regarding the morphology observed by SEM, different shapes of MOFs particles were evidenced (Figure 2). HKUST-1 (Figure 2(a1,a2)) consisted of small particles approximately 2 µm in length and 1 µm in width, along with larger particles ranging between 20–30 µm in length and 15–20 µm in width, both displaying an octahedral shape [36]. The MIL-88A material (Figure 2(b1,b2)) exhibited a distinctive morphology characterized by particles with a prismatic central body and pyramidal tips at both ends [26]. The sample consisted of well-defined elongated crystals, forming large micrometric particles measuring approximately 5.0 µm in length and 2.0 µm in width. This unique structural arrangement highlights the highly ordered structure and anisotropic crystal growth. The BASF A520 sample (Figure 2(c1,c2)) showed a morphology consisting of quasi-spherical crystals approximately 100 nm in size [37]. These nanoscale crystals likely formed as a result of rapid crystallization and were observed to aggregate into larger, micron-sized particles. This hierarchical structure reflects the interplay between crystal growth kinetics and particle assembly during synthesis. SEM images of UiO-66 (Figure 2(d1,d2)) revealed a granular morphology, characterized by aggregates of small crystallites produced through the direct reaction between zirconium salt and terephthalic acid. The particles demonstrated a homogenous size distribution around 100 nm, in agreement with previous studies reported in the literature [38]. This homogeneity underscores the precision of the synthesis process and the reproducibility of the material’s morphological features. Conversely, MOF-801 (Figure 2(e1,e2)) exhibited uniform octahedral shapes, approximately 400 nm in size.

**Figure 3:**
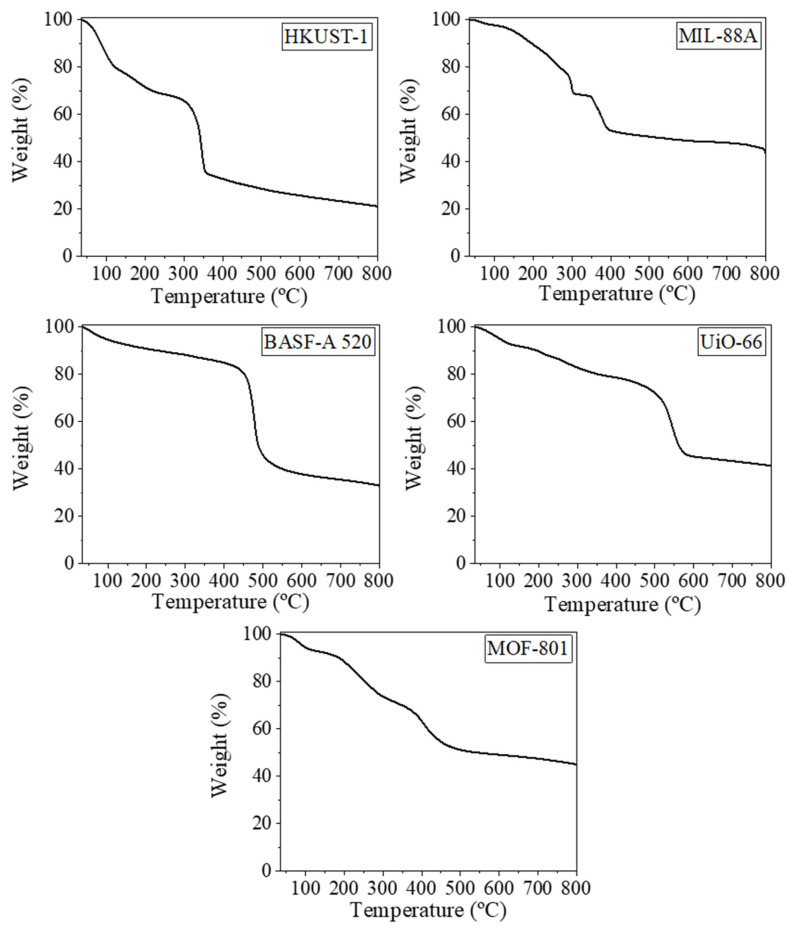
TGA curves of the MOF samples in air atmosphere.

The TGA profiles presented in Figure 3 provided valuable insights into the thermal stability of the MOFs studied. HKUST-1 displayed an initial weight loss below 200 °C, attributed to the desorption of water and residual solvents. The primary decomposition step occurs between 300 and 350 °C, corresponding to the thermal degradation of the copper-coordinated benzene tricarboxylate linkers. This behavior indicates moderate thermal stability, which is consistent with the previously reported thermal properties of copper-based MOFs [39]. MIL-88A showed an initial weight loss below 150 °C due to the release of adsorbed water and residual solvents. A significant degradation event occurred from 200 °C, associated with the breakdown of the organic linker. This thermal profile reflected the moderate thermal stability of iron-based MOFs with flexible frameworks, which is a characteristic feature of the MIL family [26]. BASF-A520, or aluminum fumarate, demonstrated a higher thermal stability compared to MIL-88A and HKUST-1. Minimal weight loss was observed below 350 °C, primarily due to solvent desorption. The major decomposition step took place between 400 and 500 °C, corresponding to the degradation of the fumarate linker. This behavior highlights the robustness of aluminum-based MOFs, which often exhibit significant thermal stability [40]. UiO-66 exhibited the highest thermal stability among the studied MOFs. Apart from minor solvent desorption below 400 °C, this material remained stable, with its primary decomposition event occurring between 450 and 550 °C. This corresponded to the breakdown of the terephthalate linker and is indicative of the exceptional thermal stability of zirconium-based MOFs, which benefit from their rigid and robust framework structure [41]. Lastly, MOF-801, another zirconium-based MOF, showed a thermal stability that was slightly lower than that of UiO-66. Negligible weight loss was observed below 300 °C, with the major degradation step occurring between 350 and 450 °C. This thermal behavior was attributed to the decomposition of the organic linker, further emphasizing the stability of zirconium-coordinated MOFs [42]. These TGA results reflect the influence of their metal centers and organic linkers on their thermal behavior, which underlines the importance of structural composition in determining the thermal properties of MOFs [43] for various applications related to active packaging.

### 2.2. Characterization of the PVA-MOF Films

**Figure 4:**
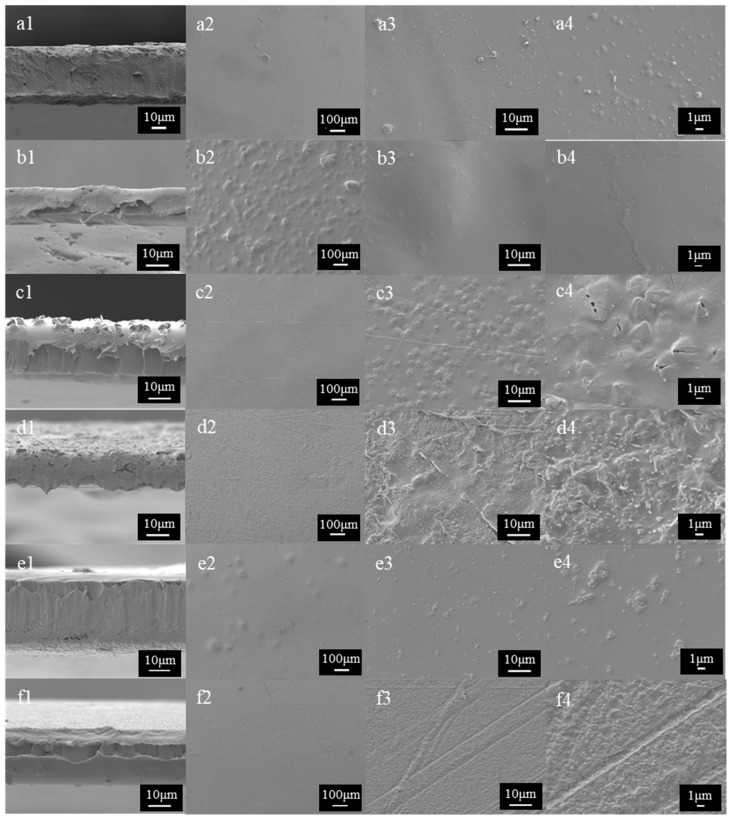
SEM images of the surface of different films obtained. These correspond to (**a1**–**a4**) PVA films, (**b1**–**b4**) PVA-HKUST-1, (**c1**–**c4**) PVA-MIL-88A, (**d1**–**d4**) PVA-BASF-A520, (**e1**–**e4**) PVA-UiO-66, and (**f1**–**f4**) PVA-MOF-801. (1) Corresponds to the cross section; (2) Corresponds to the 100× magnification; (3) Corresponds to the 1500× magnification; and (4) Corresponds to the 5000× magnification.

The morphology of the cross-section and surface of the films was examined through SEM images, shown in Figure 4. The surface images of the films demonstrated the presence of MOFs within the PVA film matrix, indicating effective homogenization, as they were distributed across the entire surface. This behavior could be expected due to the strong interaction between the MOFs and the polyhydroxylated structure of PVA [16,44,45]. However, some aggregations of MOFs were observed on the film surface, which may negatively impact the film’s properties. This phenomenon occurs because, at high concentrations, MOF particles tend to aggregate [46]. As observed in the characterization of the MOFs, these images highlighted the distinct morphology of each MOF used, both in terms of size and composition. It is also evident that there was heterogeneity in particle size among the different MOFs, as the particle size distribution results showed (Figure 1b). The cross-sectional images of the films displayed a homogeneous appearance, further indicating an effective MOF distribution throughout the matrix.

**Figure 5:**
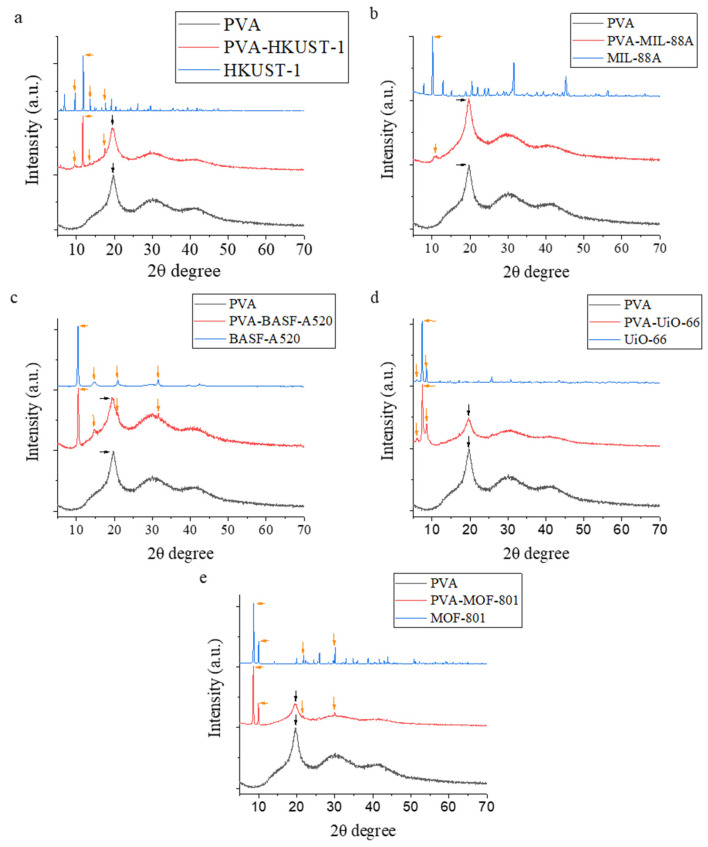
XRD pattern of the PVA-MOF composite films.

**Figure 6:**
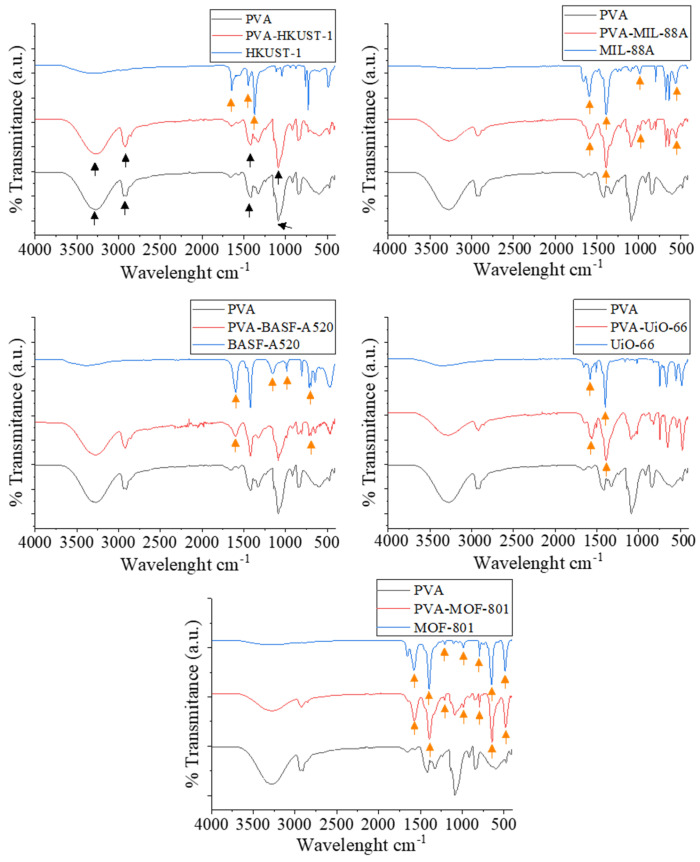
FTIR spectra of the different composite films.

The results obtained from XRD and FTIR (Figure 5 and Figure 6) confirmed the presence of the MOF in the matrix observed in Figure 4. The XRD patterns of the different composite films are shown in Figure 6. All the films showed a characteristic peak related to the semi-crystalline nature of PVA at 19.5°, attributed to the crystal plane (111) [47]. The diffraction peaks observed in the PVA-HKUST-1 film (Figure 5a) at 6.6°, 9.4°, 11.5°, and 13.3° were assigned to the (200), (220), (222) and (400) crystal planes from HKUST-1, respectively [48]. The PVA-MIL-88A film showed a diffraction peak at 10.1° which correspond to the (100) plane of the MOF (Figure 5b) [49]. The peak observed at 10.3° in PVA-BASF-A520 is related to the (100) plane of the MOF (Figure 5c) [50]. In Figure 5d, one of the most intense peaks from UiO-66 can be seen in the mixed film at 7.4° and 8.5°, which are related to the (111) and (200) crystal planes, respectively [51]. The XRD pattern of MOF-801 (Figure 5e) showed well defined peaks, which are also in the composite film. Some of them, at 10° and 21.7°, correspond to the (200) and (440) planes, respectively [52]. In the composite films, some characteristic peaks of the MOFs are clearly present in the diffractogram, although slightly attenuated, most likely due to the dominant amorphous/semi-crystalline nature of the PVA matrix and the lower relative MOF content.

The chemical structure of the films, generated from different MOFs, was studied using FTIR analysis (Figure 6). The spectra obtained from the different films showed some characteristic peaks of the PVA, such as those observed at 3285 cm^−1^, 2915 cm^−1^, 1420 cm^−1^, and 1080 cm^−1^, attributed to the stretching of -OH groups, -CH bonds, C=O and C=C bonds, and C-O bonds, respectively [53]. Some of the most characteristics peaks of HKUST-1, such as those observed at 1650, 1450, and 1365 cm^−1^, arising from asymmetric and symmetric stretching vibrations of carboxylate groups, are not observed in the PVA-HKUST-1 film [54]. The peaks observed at 1600 and 1380 cm^−1^ in the PVA-MIL-88A film correspond to symmetric and asymmetric vibration modes of the fumarate carboxyl group. Those observed at 990 and 530 cm^−1^ are attributed to C-H bond bending vibration and the stretching of the Fe-O bond, respectively [49]. In the PVA-BASF-A520 film, peaks at 690, 1400, and 1600 cm^−1^ are observed, corresponding to the Al-OH bond and symmetric and asymmetric vibrations of aluminum fumarate carboxyl groups, respectively. However, other characteristic peaks of BASF-A520 related to the O-H bond (980 cm^−1^) and C-O bond (1160 cm^−1^) cannot be observed in the film [55].The PVA-UiO-66 film presents characteristic peaks in the range of 1600–1400 cm^−1^, associated with the stretching vibrations of C=C and C=O bonds [56]. MOF-801 presents a series of characteristic peaks, all observed analogously in the film. At 1575 cm^−1^, a peak attributed to the asymmetric stretching of --C-O produced by the ligand carboxyl group can be observed. The symmetric stretching vibration of the C-H group is shown at 1400 cm^−1^. Conversely, the peaks observed at 1215, 985, and 800 cm^−1^ may be ascribed to C-O stretching, CH_3_ skeletal vibrations, and C=C-H out-of-plane bending, respectively. Additionally, the peaks at 655 and 475 cm^−1^ are indicative of vibrations associated with Zr_6_(OH)_4_O_4_ and the asymmetric stretching of the Zr-(OC) group of MOF-801 [52]. Overall, no systematic shifts were detected in the main absorption bands of PVA upon MOF incorporation, which suggests that the polymer backbone is not chemically altered. However, the attenuation or absence of some MOF-specific peaks in the composite films indicates that certain vibrational modes may be overlapped or masked by the strong absorption of PVA. While clear evidence of chemical bonding cannot be drawn from the spectra, weak physical interactions such as hydrogen bonding between PVA hydroxyl groups and MOF carboxylate or hydroxyl moieties may contribute to the compatibility observed in the films.

The physical, optical, and mechanical properties of films are critical for their application as active food packaging materials. High transparency and efficient UV dispersion enhance consumer perception by improving food visibility while protecting food from oxidative damage [57]. Strong mechanical properties are essential to withstand stress during packaging and transportation, ensuring durability while maintaining the structural integrity of the package and protecting the food [58]. Furthermore, films with enhanced barrier properties, such as lower *WVP* and reduced moisture content, are particularly desirable, as they help preserve food quality and ensure the package’s structural stability over time [59].

The results of the physical, optical, and mechanical characterization of the different PVA-MOF films are presented in Figures S1, S2 and S3, respectively, demonstrating that the addition of different MOF particles significantly influences the properties of the PVA film. Notably, distinct effects were observed among the various MOFs, attributed to their unique compositions, as well as differences in particle size, porosity, and specific surface area (Table 1 and Table 2, and Figure 1). These variations in the overall properties arise from the incorporation of MOFs into the PVA matrix, which disrupts its uniform arrangement, preventing a denser and more consistent packing of the film [60]. The MOFs with smaller particle sizes tend to be distributed more homogeneously within the matrix, resulting in a lesser impact on the film. However, larger MOF particles promote the formation of stress zones and structural discontinuities that act as weak points within the material due to their less uniform distribution [61]. These larger particles create regions with reduced cohesion between the PVA and the added material, thereby decreasing matrix uniformity and facilitating the formation of microfractures and porosity channels. This behavior explains why the density of the PVA films, as well as the transparency of the films, decreases upon the incorporation of MOFs, with films containing smaller average particle sizes (such as MIL-88A and MOF-801) being less affected. Similarly, the films’ moisture content was reduced due to the strong interactions between MOFs and the PVA polymer matrix [16], which limit water uptake by preventing hydrogen bonding with the PVA matrix. However, MOFs with higher porosity, such as HKUST-1, may allow for slightly more interactions with water molecules.

Another aspect to consider is the stability in aqueous media that MOFs present. This stability depends on several factors, including the nature of the metal center, the type of ligand, and the interaction between them. Metals with higher valence states, such as Zr^4+^, tend to form stronger bonds with carboxylate groups due to their higher charge density, which allows for more stable coordination with the ligands. This results in a more robust structure that is less susceptible to hydrolysis. In contrast, carboxylate ligands, when coordinated with lower-charge metals like copper (Cu^2+^), form weaker bonds, making these MOFs more prone to decomposition and dissolution in water [62]. These findings are consistent with the results obtained for water solubility, in which HKUST-1 (Cu^2+^) exhibited significantly higher values compared to the others. On the other hand, the PVA-MIL-88A and PVA-A520-BASF films exhibited intermediate behavior as they do not interact as strongly as those with metals of higher valence states, like zirconium. Furthermore, particle size also affects solubility. Larger MOF particles, such as those in HKUST-1, facilitate water access to the PVA matrix by creating microfractures and porosity channels, thereby increasing overall solubility. The low water stability of HKUST-1, as well as the size of its particles and the associated issues, would explain why it exhibits higher *WVP* values compared to the other films.

The films containing MOFs exhibited a significant improvement in UV blocking properties compared to the PVA film. This behavior has been previously observed with various MOFs in prior studies and is attributed to proper particle dispersion within the matrix, good interaction with the matrix, their composition (metal center and ligand), and properties that enable UV light reflection (porous structure and surface area) [44,63,64].

Previous studies have reported improvements in the mechanical properties of PVA films with the addition of MOFs, likely due to strong interactions with the polyhydroxylated structure of the PVA matrix [16,44,45]. However, the results in Figure S3 deviate from these expectations, as most films showed no significant differences in mechanical properties, and in some cases, even a notable decrease is observed. This may be because prior studies achieved improvements using a lower MOF content (typically 0.5–2% by weight) [46]. As observed in the SEM images (Figure 4) and noted in previous sections, the MOF particle size and its tendency to aggregate play a critical role in influencing film properties. Smaller MOF particles were more uniformly dispersed in the PVA matrix [65], reducing the formation of microfractures and porosity channels. Conversely, larger MOF particles with higher aggregation tendencies create stress zones that weaken the structure. This is consistent with the mechanical characterization results, in which films with smaller-sized MOFs (MIL-88A and MOF-801) exhibit greater tensile strength and deformation resistance.

### 2.3. Characterization of the CNFs

Table 3 presents the results of the characterization of the cellulose nanofibers obtained from cotton linters, as well as data from other raw materials processed using similar methods. The results of the cotton linter nanofibers exhibit values that are very similar, and in some cases even superior, to those of the other nanofibers in terms of cationic demand, with the exception of those derived from orange peels. Specifically, the results closely resemble those of wheat straw nanofibers, which are commonly used as reinforcing agents in PVA films. This similarity is particularly evident in parameters such as cationic demand, specific surface area, and diameter. This suggests that cotton linter nanofibers could be suitable for use as reinforcing agents in PVA films. Therefore, incorporating these nanofibers could not only enhance the mechanical and barrier properties of PVA-CNF films but also facilitate a more uniform distribution of MOFs throughout the matrix. This improved distribution and enhanced performance make these films an even more promising material for active packaging applications. Additionally, the addition of CNF potentially could improve the overall distribution throughout the film by reducing MOF aggregation at high concentrations and thus leading to an overall improvement in properties [18].

### 2.4. Characterization of the PVA-CNF-MOF Films

#### 2.4.1. PVA-CNF Films

To assess the reinforcement capacity of our nanofibers, multiple PVA-CNF films were prepared at various nanofiber concentrations. The results are shown in Table S1.

The evaluation of mechanical properties reveals that both the tensile strength and Young’s modulus increase as the CNF content rises, with a significant peak observed at the 5% incorporation level. This enhancement is largely attributed to the nanofibers’ ability to create inter- and intramolecular hydrogen bonds, their more uniform dispersion within the matrix, and their high compatibility with the PVA. Nevertheless, when the CNF concentration surpasses 5%, the films experience a reduction in tensile strength and Young’s modulus. This decline is likely caused by the tendency of nanofibers to aggregate at higher concentrations, leading to the formation of clusters within the matrix, which in turn weakens the material [23].

The *WVP* results indicate a significant improvement in the barrier properties of the films after the incorporation of nanofibers, with values decreasing from 0.1749 ± 0.0109 for pure PVA to 0.0622 ± 0.0062 (g·Pa^−1^·s^−1^·m^−2^·10^−7^) for the 5% CNF composition. This effect is attributed to the so-called tortuous pathway, where the nanofibers act as fillers within the matrix, obstructing the optimal movement of water molecules, which become trapped. The films with the CNF show better results, as previously mentioned, due to a more homogeneous nanofiber distribution within the matrix, further enhancing the barrier properties. However, higher nanofiber concentrations lead to an expected increase in *WVP*, likely due to the formation of nanofiber aggregates that facilitate the passage of water molecules [23,69]. This behavior was expected to result in a lower moisture content, as well as a reduction in solubility and swelling. However, no significant differences were observed in moisture content, and a slight increase in swelling and moisture was noted in some film compositions. The transparency of the films is also related with the dispersion of the nanofibers in the PVA matrix [67]. As shown in the results, an increase in the nanofiber content leads to a decrease in transparency, indicating a homogeneous dispersion within the film. However, the 7% composition shows an increase in transparency, which may be due to the aggregation of nanofibers, facilitating the passage of light. The UV barrier results did not show any significant difference between the different films.

Based on the results obtained, it was determined that the composition with 5% cellulose nanofibers exhibited the greatest improvement in properties relevant to active packaging, such as *WVP* and overall mechanical properties. Therefore, this composition was selected for the fabrication of the PVA-CNF-MOF films.

#### 2.4.2. Characterization of the PVA-CNF-MOF Films

**Figure 7:**
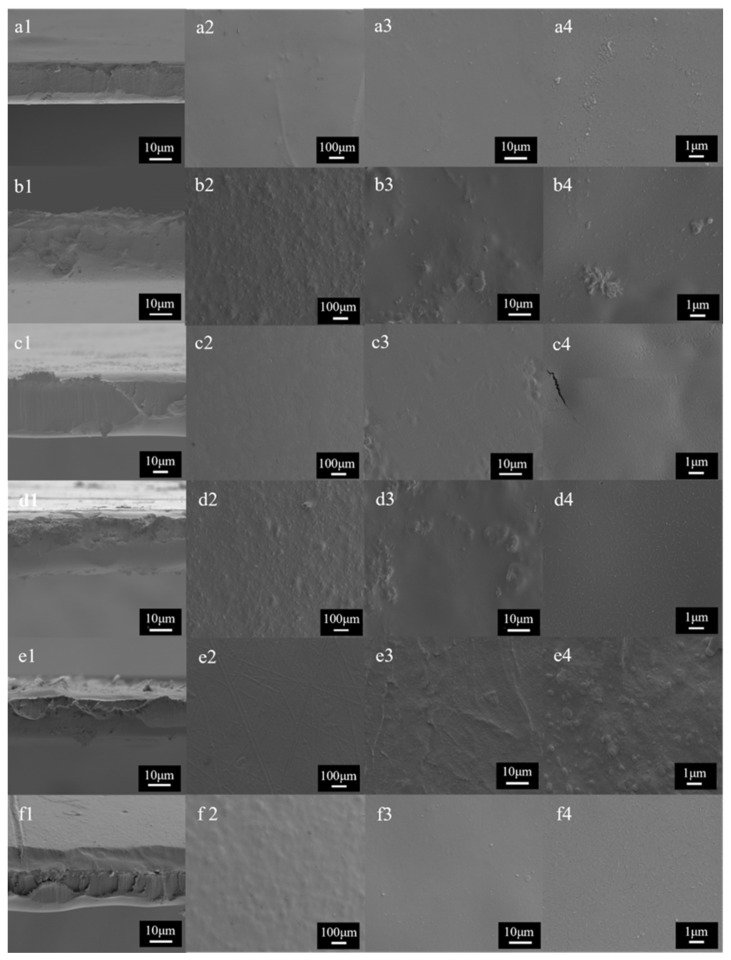
Surface images of various films obtained via SEM. These correspond to (**a1**–**a4**) PVA-CNF, (**b1**–**b4**) PVA-CNF-HKUST-1, (**c1**–**c4**) PVA-CNF-MIL-88A, (**d1**–**d4**) PVA-CNF-BASF-A520, (**e1**–**e4**) PVA-CNF-UiO-66, and (**f1**–**f4**) PVA-CNF-MOF-801 films. (1) Corresponds to the cross-section view; (2) Corresponds to the 100× magnification; (3) Corresponds to the 1500× magnification; and (4) Corresponds to the 10,000× magnification.

Figure 7 shows SEM images of the cross-section and surface of the PVA-CNF-MOF films. These images are significantly different from the images obtained for the PVA-MOF films (Figure 4). A more homogeneous distribution and the absence of aggregations are evident on the film surfaces, even at higher magnifications. Similarly, the cross-section of the films presents a homogeneous appearance, further indicating effective MOF distribution throughout the matrix. This behavior is expected, as previously mentioned, since cellulose nanofibers enhance the dispersion of MOFs due to the strong interaction between the carboxylate groups of the nanofibers and the metal centers of the MOFs, serving as binding sites for the MOFs [18]. This is a crucial aspect, as it is expected to result in the improved physical and mechanical properties of the films.

The XRD and FTIR results are shown in Figures S4 and S5, respectively. In the FTIR spectrum, no characteristic peaks of cellulose were observed due to overlapping with the characteristic peaks of PVA, attributed to their similar structure and the low concentration of cellulose nanofibers present [70]. However, the presence of cellulose nanofibers can be identified in the XRD spectrum, which shows a diffraction peak at 22°, corresponding to the (200) crystallographic plane of cellulose [71]. In both spectra, the characteristic peaks of the respective MOFs, as well as those of PVA, are also observed, as previously described.

**Figure 8:**
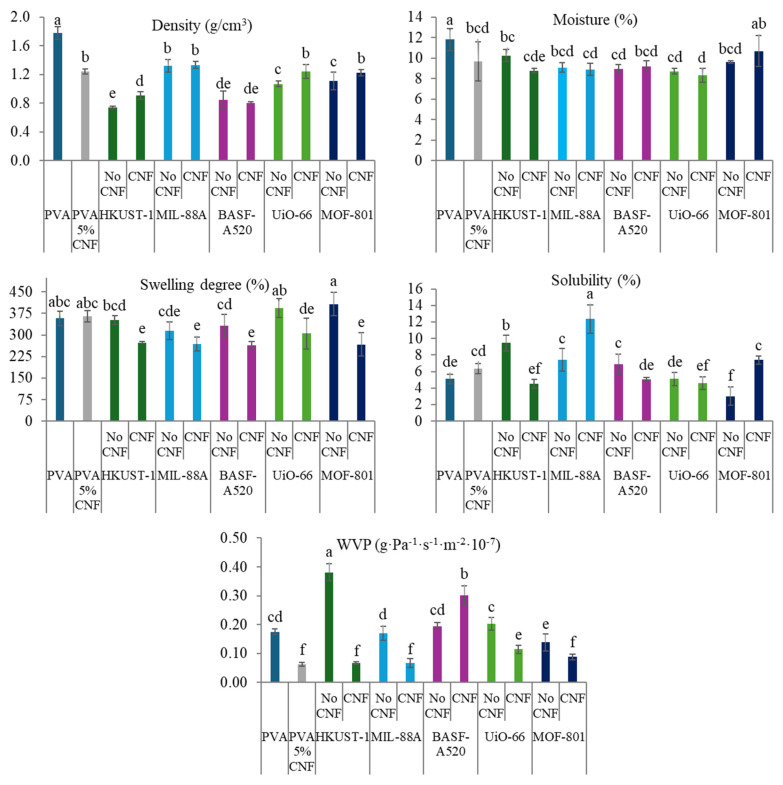
Physical properties of the PVA-MOF and PVA-CNF-MOF films. Different letters above bars indicate significant differences among formulations (*p* ≤ 0.05).

**Figure 9:**
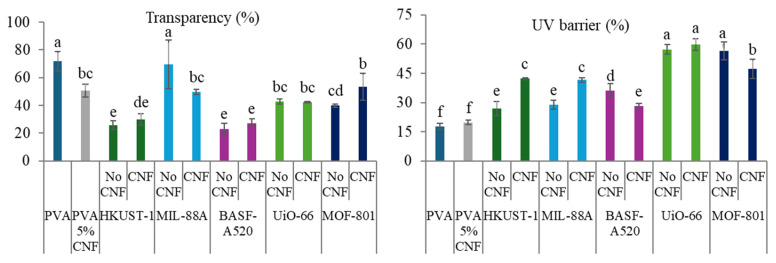
Optical properties of the PVA-MOF and PVA-CNF-MOF films. Different letters above bars indicate significant differences among formulations (*p* ≤ 0.05).

**Figure 10:**
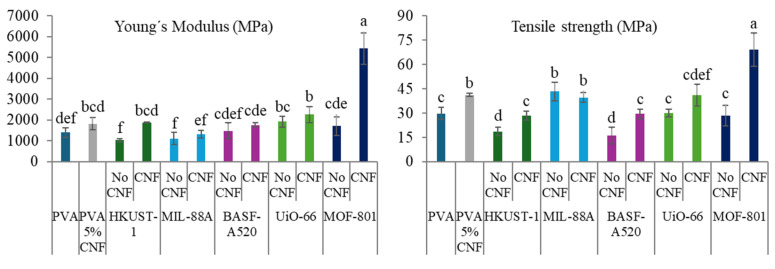
Mechanical properties of the PVA-MOF and PVA-CNF-MOF films. Different letters above bars indicate significant differences among formulations (*p* ≤ 0.05).

The expected improvement in the properties of the films with the inclusion of CNF is evident in the results obtained from the characterization of their physical, optical and mechanical properties (Figure 8, Figure 9 and Figure 10). As previously mentioned, and as observed in the SEM images (Figure 7), the presence of cellulose nanofibers likely facilitates a more homogeneous dispersion of MOFs within the matrix. This prevents the formation of stress zones and structural discontinuities that could act as weak points in the material [61], while compacting the matrix, thereby reducing both porosity and accessibility. This phenomenon explains the slight increase in density and in mechanical properties (Figure 10), as well as the significant reduction in swelling values and *WVP* (Figure 8), which is also associated with the tortuous pathway mechanism previously described [23]. Additionally, films containing MOFs (HKUST-1, BASF-A520, and UiO-66) with larger particles showed a notable reduction in solubility values, as expected. Conversely, an increase in solubility is observed in films incorporating smaller MOFs. These unexpected behaviors could be attributed to the film formation process itself.

The improved distribution provided by the CNF is also reflected in the optical properties (Figure 9). The enhanced dispersion of the MOFs results in a more uniform distribution on the film surface, which explains the observed variations compared to the films without CNF.

The mechanical properties of the films, shown in Figure 10, exhibit a significant improvement with the incorporation of cellulose nanofibers, particularly highlighting the reinforcing effect in compositions containing HKUST-1 and MOF-801. The film with MOF-801 achieved exceptional values for its Young’s modulus and tensile strength, reaching 5426 MPa and 69.12 MPa, respectively. These values represent an increase of approximately 290% in its Young’s modulus and 130% in its tensile strength compared to the properties of pure PVA.

### 2.5. Scavenging Activity

The results obtained from the proof-of-concept test to measure ethylene retention by the PVA-MOF and PVA-CNF-MOF films are shown in Table 4. This table presents the texture and browning rate results for the selected bananas. The texture results demonstrate the mechanical strength of the banana pulp after being stored in a controlled environment with the films. Some compositions showed significant improvements with the incorporation of CNF, such as in the case of HKUST-1, BASF-A520, and MOF-801, highlighting the importance of proper homogenization to improve the active surface of the films. Similarly, the browning rate also showed improvements in films with CNF compared to their counterparts without nanofibers. However, despite the observed improvements in films incorporating CNF and the different ethylene adsorption capacities of the MOFs (Table 1), only small differences were observed between them and the PVA film, in which the difference in the firmness (texture) of the bananas was notable.

In future studies, using freshly harvested fruit under controlled storage conditions could help reduce variability and provide a clearer assessment of the effect of MOFs on the formulations. The variability observed in this study may be attributed to the natural heterogeneity of fresh produce, particularly when sourced from supermarkets. Differences in cultivation practices, prior storage conditions, environmental exposure, and handling can influence key parameters such as texture and browning rate, potentially masking the impact of MOFs on fruit preservation [72,73,74,75].

Nevertheless, PVA-CNF-MOF-801 films show great promise due to their excellent mechanical properties, high UV protection, and low *WVP*. The superior mechanical performance observed for PVA-CNF-MOF-801 films can be attributed to the intrinsic stability of the MOF-801 framework, its smaller particle size, and its favorable interaction with the polymeric and CNF matrix. These factors likely promoted better interfacial adhesion and stress transfer compared with other MOFs, thereby enhancing their tensile strength and Young’s modulus. Additionally, PVA-CNF-MIL-88A films are also of interest, despite their mechanical properties being inferior to those of PVA-CNF-MOF-801 films. Both MOFs exhibit high biocompatibility, making them suitable for direct contact with food products.

## 3. Materials and Methods

### 3.1. Materials

Cellulose derived from cotton linter was supplied by Cotton South S.L (Granada, Spain). Sodium bromide (NaBr, 99% purity) was supplied by ACS reagent (Washington, DC, USA). Ethanol was supplied from Alcoholes del Sur S.A (Córdoba, Spain). Cupric nitrate hemipentahydrate (Cu(NO_3_)_2_·2.5H_2_O), benzene-1,3,5-tricarboxylic acid, iron(III) chloride hexahydrate (FeCl_3_·6H_2_O), fumaric acid, aluminum sulfate hydrate (Al_2_(SO_4_)_3_·18H_2_O), zirconium(IV) chloride (ZrCl_4_), 1,4-benzenedicarboxylic acid, zirconium oxychloride octahydrate (ZrOCl_2_·8H_2_O, purity ≥ 99.5%), formic acid, N,N-Dimethylformamide anhydrous (DMF, purity ≥ 99.8%), 2,2,6,6-tetramethylpiperidine-1-oxyl (TEMPO, free radical, 98% purity), Bis-(ethylenediamine)copper(II) hydroxide solution (CED), and poly(vinyl alcohol) (PVA) were supplied by Sigma Aldrich (St. Louis, MO, USA). Sodium hypochlorite solution (10% *w*/*v*, technical grade) and sodium hydroxide (pellets technical grade) were supplied from PanReac AppliChem ITW Reagents (Barcelona, Spain). Pes-Na (0.001 N) and Cationic Titrant (0.001 N) were provided by BTG Instruments AB (Espoo, Finland). Granulated calcium chloride anhydrous (extra pure) from Scharlau Chemie S.A (Barcelona, Spain) was employed.

All MOF materials were prepared following procedures previously reported in the literature: HKUST-1 [76], MIL-88A [26], BASF-A520 [31], UiO-66 [77], and MOF-801 [78].

### 3.2. Synthesis of the TEMPO-Oxidized Cellulose Nanofibers (CNFs)

Cellulose nanofibers were obtained by chemical pretreatment process involving TEMPO catalytic oxidation and subsequent high-pressure homogenization. This pretreatment involves the oxidation of cellulose through the addition of 5 mmol NaClO/g fiber in the presence of NaBr (0.1 g/g fiber) and TEMPO (0.016 g/g fiber), followed by the addition of 0.5 M NaOH until the reaction reaches stable pH values of 10.2. Once the reaction time is completed (2 h), 100 mL of ethanol was added, the pulp was filtered and a 1.5% suspension in distilled water was prepared and stirred for 24 h. After this time, high-pressure homogenization was carried out using an optimized pass sequence [67] in the GEA Niro Panda Plus 2000 high-pressure homogenizer (GEA Spain Alcobendas, Alcobendas, Madrid, Spain). The nanofibers generated were stored under refrigeration at 4 °C.

### 3.3. Synthesis of the PVA-CNF-MOF Films

Films with a grammage of 35 g/m^2^ were fabricated using a solvent casting technique. For this purpose, an aqueous solution of PVA was prepared at a concentration of 30 g/L. The mixture was then stirred for 3 h at 85 °C.

To create MOF/PVA films, different types of MOFs were added up to 10% with respect to the dry weight of PVA. The MOF/PVA mixtures were then diluted to a total volume of 100 mL and mechanically agitated for 3 h to ensure proper homogenization. After the time elapsed, the solutions were poured into Petri dishes with an area of 144 cm^2^ and dried in a climate-controlled environment at 25 °C with 50% relative humidity.

The reinforcing effect of CNF on PVA films was investigated by adding different proportions of nanofibers (1, 3, 5, and 7% based on the dry weight of PVA). For this purpose, the determined amount of CNF was diluted in 50 mL of distilled water and dispersed by applying a homogenizer IKA T18 digital Ultra Turrax (IKA Works Spain, Limited Company, Barcelona, Spain). Once uniformly dispersed, they were mixed with the predetermined amount of PVA, further diluted to 100 mL, shaken and then poured into Petri dishes.

MOF-PVA-CNF films with the optimal nanofiber content were prepared following a similar procedure as described above. In this case, CNFs were pre-mixed with the various MOFs at different concentrations (1, 3, 5, and 10% *w*/*w* based on PVA) and homogenized prior to blending with PVA. All film preparations were conducted in triplicate to evaluate their reproducibility.

**Figure 11:**
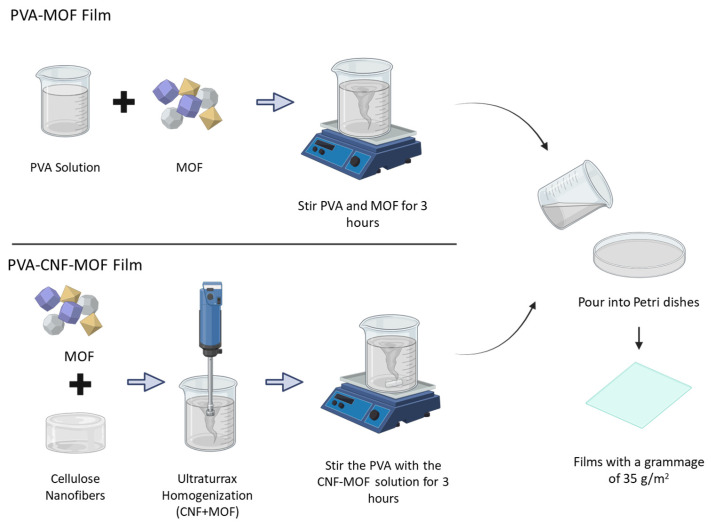
Step-by-step schematic of the synthesis procedures for PVA-MOF and PVA-CNF-MOF films.

A schematic illustration summarizing the workflow for producing PVA-MOF and PVA-CNF-MOF films is provided in Figure 11.

### 3.4. Structural, Textural, Chemical and Morphological Characterization Techniques

X-ray diffraction (XRD) patterns were obtained using a Bruker D8 Discover diffractometer (Bruker, Berlin, Germany) equipped with a LynxEye linear position-sensitive detector and a Cu-Kα radiation source (λ = 1.5418 Å), operating in reflection geometry mode. A Ni filter was used to suppress the Kβ radiation, and both Kα_1_ and Kα_2_ components were included in the analysis. The scans were performed over a 2θ range of 5–70°, with a step size of 0.02°, at a scan rate of 0.04°/min, using fixed divergence and receiving slits. The total acquisition time per sample was approximately 55 min. Samples were mounted on standard Bruker polymer-based transparent sample holders, which are optimized for thin film analysis and provide minimal background signal. The textural properties of the MOFs were analyzed through N_2_ adsorption-desorption isotherms in a Micromeritics ASAP 2020 instrument (Micromeritics, Norcross, GA, USA) at −196 °C. Before the measurements, a degasification step was carried out at 120 °C overnight under vacuum conditions (10^−6^ mbar) to remove residual adsorbed gases and impurities. Thermogravimetric analysis (TGA) measurements were performed using a Mettler Toledo-TGA/DSC (Mettler Toledo, Greifensee, Switzerland) under air atmosphere by heating the samples from 25 to 800 °C at 10 °C min^−1^. Particle size analysis was performed using a Mastersizer S laser diffraction system (Malvern Instruments, Malvern, UK). The sample was dispersed in ethanol and subjected to ultrasonic treatment for 10 min to prevent particle aggregation. Volume Moment Mean (D [4.3], De Brouckere Mean Diameter) was obtained for each MOF material. The incorporation of MOFs and the CNF in the films was evaluated by Fourier transform infrared spectroscopy (FTIR) using a Perkin-Elmer Spectrum Two FTIR-ATR spectrometer (PerkinElmer U.S. LLC, Shelton, CT, USA). The spectrum was analyzed with a resolution of 4 cm^−1^ in a range of 400–4000 cm^−1^. The different morphologies of the MOFs, PVA-MOF films, and PVA-CNF-MOF films were examined by Scanning Electron Microscopy (SEM) technique on a JEOL JSM 7800 F Scanning Electron Microscope (JEOL Ltd., Akishima, Japan). For this purpose, the samples were coated with gold to improve the conductivity. The accelerating voltage applied to the samples was 5 KV, with a working distance of 10 mm.

### 3.5. Characterization of the CNFs

The CNFs were characterized in terms of their nanofibrillation yield, cationic demand, carboxylation rate, and degree of polymerization. The nanofibrillation yield was determined by preparing a 0.1% suspension of the nanofibers, followed by centrifugation for 12 min at 10,000 rpm. For the cationic demand determination, 0.1% suspension was prepared with 0.2 dry grams. Once homogenized, a 15 mL aliquot was mixed with 15 mL of a cationic polymer (cationic titrant) and centrifuged at 4000 rpm for 90 min. Subsequently, a Mütek PCD 05 particle charge detector (Malvern Panalytical Ltd., Malvern, UK) was used to determine the cationic demand of the supernatant aliquots.

The carboxyl content was calculated by conductimetry of a 0.3 g dry sample of nanofiber, diluted to 55 g with distilled water and adding 5 mL of 0.1 M NaCl to increase the conductivity, followed by titration with a standardized 0.01 M NaOH solution.

The results obtained for the carboxyl rate and cation demand were used for the calculation of the specific surface area (*σ*) and the diameter of the nanofibers (*d*), making use of results and equations reported in the literature (Equations (1) and (2)) [79]:

(1) $$\sigma_{nanofibers}(m^{2}/g)=\left(CD-CC\right)\cdot \sigma_{cationic\;titrant}$$

where *CD* is the cationic demand and *CC* is the carboxyl content.

(2) $$d(\mathrm{nm})=\frac{4}{\sigma_{nanofibers}\cdot 1600\cdot 10^{3}}$$

The measurement of the degree of polymerization (*DP*) was determined by calculating the intrinsic viscosity according to [80]. For this purpose, a given amount of nanofiber was dissolved in 50 mL of 0.5 M CED solution and measured using a Manual SCAN/ISO 5351 Viscometers Type T (PSL Rheotek, Granger, IN, USA). The results of the degree of polymerization were used to calculate fiber length using the following equation (Equation (3)) [81]:

(3) $$Length\;\left(\mathrm{nm}\right)=4.286\cdot DP-757$$

### 3.6. Mechanical Properties of the PVA-CNF-MOF Films

Young’s modulus and tensile strength of the films were determined using an LF Plus Lloyd Instrument testing machine with a 1 kN cell load. The analyses were performed on film samples measuring 10 cm in length and 1.5 cm in width accordance with ASTM D882. For each sample the test was repeated 10 times, and the results are expressed as the mean value accompanied by its corresponding standard deviation.

### 3.7. Physical Properties of the PVA-CNF-MOF Films

The films obtained were measured in terms of weight, area and thickness to calculate the density of the films. The moisture content of the films was calculated by determining weight differences of the films before and after putting them in an oven at 105 °C for 24 h. These same fragments are used to determine the swelling and water solubility of the films. To determine swelling, the dried fragments were placed in 50 mL of distilled water for 24 h. After this time, they were extracted, the excess water was removed by depositing them on a filter paper, and the weight was recorded. These fragments were returned to the oven for 24 h to calculate the water solubility. All these measurements were carried out in triplicate, and their result is shown as the mean with the standard deviation.

Water vapor permeability (*WVP*) of the films was determined according to ASTM E96/E96M-10 standard test method. For this purpose, TQC SHEEN Permeability Cup aluminum capsules with a surface area of 10 cm^2^ were used. The capsules were loaded with CaCl_2_ to serve as the desiccant material. These capsules, containing the film and salt, were then introduced into a climatic chamber at 25 °C and 50% RH, where the weight was recorded at different hours. The *WVP* result was calculated using the following equations (Equations (4) and (5)):

(4) $$WVP=\frac{WVTR}{∆p}=\frac{WVTR}{S(R_{1}-R_{2})}$$

In this equation, ∆p represents the vapor pressure difference, *S* denotes the saturation vapor pressure at the test temperature, and *R*_1_ and *R*_2_ indicate the relative humidities at the source and vapor sink, respectively, presented as fraction.

(5) $$WVTR=\frac{G}{tA}$$

where *G* is the weight variation (g), *t* is the time elapsed during the measurement (h), and *A* is the test area (ft^2^).

### 3.8. Optical Properties of the PVA-CNF-MOF Films

The ultraviolet barrier and transparency of the films were determined by measuring their transmittance at 280 and 660 nm, respectively, using a Jenway 7315 Advance UV/Visible Spectrophotometer (Fisher Scientific International, Inc., Hampton, VA, USA). The transmittance (*T*) values were then processed using the following equations (Equations (6) and (7)):

(6) $$Transparency\;\left(\%\right)=\;\frac{log\%T_{660}}{thickness\;(\mathrm{mm})}$$

(7) $$UV-light\;barrier\;\left(\%\right)=100-\left(\frac{\%T_{280}}{\%T_{660}}\cdot 100\right)$$

### 3.9. Ethylene Scavenging Application

The ethylene retention capacity of the films with 10% MOF were tested through an experiment using bananas. Bananas from the same bunch were selected and placed in zip-lock polyethylene bags, with films inside. The bags containing the bananas were stored in a climatic chamber at 25 °C and 50% relative humidity for eight days. Throughout the experiment, the bananas were photographed daily to monitor the progression of browning, which was quantified using the following equation (Equation (8)):

(8) $$R_{b}=S_{b}/S_{t}$$

where *S_b_* represents the brown area, and *S_t_* is the total area of the banana peel [82].

The firmness of the bananas was measured on the ninth day using a TA. XT Plus texture analyzer equipped with a P6 cylindrical probe (6 mm diameter). Samples with a thickness of 1.5 cm were taken from different parts of the banana (front, rear, and middle sections), and measurements were performed in triplicate. The test was conducted with a testing speed of 2.00 mm/s, a post-test speed of 2.00 mm/s, and a plotting parameter of 5.00 mm/s over a distance of 5 mm [83].

### 3.10. Statistical Analysis

The experimental results are expressed as the mean ± standard deviation. Analysis of variance (ANOVA) followed by the Duncan post hoc test were performed. Samples were considered to be statistically different at *p* ≤ 0.05.

## 4. Conclusions

This study evaluated the potential of PVA-MOF and PVA-MOF-CNF films for active packaging applications. The incorporation of TEMPO-oxidized cellulose nanofibers (CNFs) significantly enhanced the homogeneity of the MOF distribution, reducing aggregation and improving the interaction between components. This improvement led to a more structurally and functionally cohesive matrix, resulting in a superior overall performance compared to their counterparts without nanofibers. Notably, the films exhibited substantial improvements in mechanical properties, including significant increases in Young’s modulus and tensile strength. Additionally, the barrier properties were enhanced, with a marked reduction in *WVP* and improved UV shielding, which was attributed to the MOFs. These enhancements are critical for ensuring the performance of the films in protecting and extending the shelf life of perishable foods, underscoring their suitability for active packaging applications.

While the PVA-CNF-MOF films showed improved ethylene retention compared to PVA-MOF films, the differences in ethylene retention among the PVA-CNF-MOF variants were statistically very small. This limitation highlights the influence of external factors on product variability, emphasizing the need for subsequent research to refine experimental conditions and isolate the effects of the materials themselves. To address this, future studies should consider using freshly harvested fruit stored under controlled conditions to minimize variability and provide a clearer assessment of MOF performance.

Despite this limitation, PVA-CNF-MOF-801 demonstrated the most promising results among the compositions tested in the ethylene scavenging analysis. This film exhibited high biocompatibility, making it suitable for direct contact with food products, in this case, bananas. This characteristic, combined with their enhanced mechanical and barrier properties, reinforces the potential of PVA-CNF-MOF films as viable materials for food packaging applications. In the future, their use could be expanded to other ethylene-producing commodities, such as tomatoes, avocados, or apples, thereby contributing to sustainable strategies for extending the shelf-life of fresh produce.

## Figures and Tables

**Table 1:** Summary of structural and adsorption properties of the MOFs synthesized for this study, highlighting their potential as candidates for ethylene capture based on prior studies [26]. BTC = 1,3,5-benzenetricarboxylic acid, Fum = Fumaric acid, BDC = 1,4-benzenedicarboxylic acid.

Material	Metallic Center	Organic Linker	S_BET_ (m^2^/g)	Theoretical Pore Size (Å)	Ethylene Adsorption (mmol·g^−1^)
HKUST-1	Cu	BTC	2042	9.0	8.33
MIL-88A	Fe	Fum	359	6.0	1.63
BASF-A520	Al	Fum	911	5.8	3.90
UiO-66	Zr	BDC	1400	7.5, 12.0	2.61
MOF-801	Zr	Fum	856	4.8, 5.6, 7.4	2.31

**Table 2:** Porous textural properties of MOF samples.

MOF Sample	Langmuir Surface Area (m^2^/g)	t-Plot Micropore Area (m^2^/g)	t-Plot External Surface Area (m^2^/g)	Total Pore Volume (cm^3^/g)	t-Plot Micropore Volume (cm^3^/g)	BJH Desorption Average Pore Width (nm)
HKUST-1	1526	1471.8	54.2	0.584	0.512	4.8
MIL-88A	469	429.9	39.4	0.198	0.146	4.9
BASF-A520	968	909.2	59.2	0.414	0.313	10.6
UiO-66	1531	1478.0	52.6	0.585	0.514	6.3
MOF-801	853	797.1	55.6	0.333	0.272	4.9

**Table 3:** Results from the characterization of the TEMPO nanocellulose compared with other CNFs obtained from different sources.

Parameters	Yield (%)	Cationic Demand (µeq/g)	Carboxyl Content (µeq/g)	Specific Surface Area (m^2^/g)	Diameter (nm)	Length (nm)
Cotton Linter (This work)	63.55 ± 7.89	1178.59 ± 59.02	534.02 ± 27.61	314 ± 32	8.01 ± 0.82	319.29 ± 30.66
Raspberry [57]	˃95	1264 ± 32.6	240.2 ± 4.5	548	5	N.R.
Horticultural residues (bell pepper, tomato, eggplant) [66]	63.44 ± 4.52	1043.54 ± 18.2	148.12 ± 5.26	436	6	614
Vine shoots [66]	60.42 ± 5.56	1227.91 ± 18.8	168.93 ± 10.25	516	5	755
Orange peel wastes [23]	75.58 ± 2.21	2144.73 ± 29.64	559.73 ± 16.60	772	3	2383
Wheat straw [67]	98.71	1116.5 ± 43.10	367.0 ± 8.72	367.01	6.81	1395
Olive tree pruning [68]	26.44 ± 4.15	521.27 ± 9.33	311.95 ± 19.02	101.93	24	705

**Table 4:** Results of browning rate and texture obtained from the banana characterization. Different letters under texture and browning rate parameters indicate significant differences among formulations (*p* ≤ 0.05).

Parameters	Texture (g)		Browning Rate (%)	
Samples	PVA-MOF	PVA-CNF-MOF	PVA-MOF	PVA-CNF-MOF
0% MOF	77.80 ± 33.09 bcd	64.81 ± 22.29 de	40.82 ± 13.81 bc	44.37 ± 7.69 b
HKUST-1	53.19 ± 20.80 e	77.02 ± 35.47 bcd	37.01 ± 7.94 bcd	20.28 ± 0.00 d
MIL-88A	78.11 ± 21.56 bcd	71.97 ± 25.67 bcd	42.06 ± 7.78 bc	37.30 ± 1.96 bcd
BASF-A520	67.13 ± 19.75 cde	85.91 ± 16.19 ab	41.88 ± 18.70 bc	26.69 ± 4.66 bcd
UiO-66	98.11 ± 22.94 a	83.32 ± 21.56 abc	33.64 ± 7.04 bcd	28.33 ± 17.25 bcd
MOF-801	63.91 ± 16.41 de	87.15 ± 26.82 ab	64.29 ± 16.56 a	22.53 ± 29.50 cd

## Data Availability

The data underlying this study are fully presented within the manuscript. Further information or specific datasets used in the analysis are available from the corresponding author upon request.

## Supplementary Materials

The following supporting information can be downloaded at: https://www.mdpi.com/article/10.3390/molecules30193971/s1, Figure S1: Physical properties (density, moisture, swelling degree, solubility and *WVP*) of the composite films. Different letters above bars indicate significant differences among formulations (*p* ≤ 0.05); Figure S2: Transparency and UV barrier of the different composite films. Different letters above bars indicate significant differences among formulations (*p* ≤ 0.05); Figure S3: (a) Young’s Modulus, (b) tensile strength and (c) stress-strain curves of PVA films with the different MOFs. Different letters above bars indicate significant differences among formulations (*p* ≤ 0.05); Figure S4: XRD pattern of the PVA-CNF-MOF films; Figure S5: FTIR of the PVA-CNF-MOF composite films; Table S1: Results of the characterization of the PVA-CNF composite films in terms of mechanical, physical and optical properties.
