# Supplementary material for: Engineering PVA-CNF-MOF Composite Films for Active Packaging: Enhancing Mechanical Strength, Barrier Performance, and Stability for Fresh Produce Preservation
Source: Molecules. 2025 Oct 3;30(19):3971. doi: 10.3390/molecules30193971 (PMC12526449; doi:10.3390/molecules30193971)
Supplement: Supplementary file 1 [file molecules-30-03971-s001.zip › molecules-3804262-supplementary.pdf]

SUPPLEMENTARY INFORMATION

Engineering PVA-CNF-MOF Composite Films for Active Packaging: Enhancing Mechanical Strength, Barrier Performance, and Stability for Fresh Produce Preservation

Sergio Carrasco [1], Juan Amaro-Gahete [2,3,*], Eduardo Espinosa [1], Almudena Benítez [4], Francisco J. Romero-Salguero [3] and Alejandro Rodríguez [1]

[1] BioPrEn Group (RNM940), Chemical Engineering Department, Instituto Químico para la Energía y el Medioambiente (IQUEMA), Faculty of Science, University of Córdoba, 14014 Córdoba, Spain; q62carcs@uco.es (S.C.); a02esvie@uco.es (E.E.); q42ropaa@uco.es (A.R.)

[2] Materiales Polifuncionales Basados en Carbono (UGR-Carbon), Department of Inorganic Chemistry, Unidad de Excelencia Química Aplicada a Biomedicina y Medioambiente, University of Granada (UEQ-UGR), 18071 Granada, Spain

[3] Deparment of Organic Chemistry, Instituto Químico para la Energía y el Medioambiente (IQUEMA), Faculty of Science, University of Córdoba, Campus de Rabanales, Ed. Marie Curie, 14071 Córdoba, Spain; qo2rosaf@uco.es

[4] Departament of Inorganic Chemistry and Chemical Engineering, Instituto Químico para la Energía y el Medioambiente (IQUEMA), Faculty of Science, University of Córdoba, 14071 Córdoba, Spain; q62betoa@uco.es

* Correspondence: j.amarogahete@ugr.es

Table S1. Results of the characterization of the PVA-CNF composite films in terms of mechanical, physical and optical properties.

Parameters	Density (g/cm^3)	Moisture (%)	Swelling degree (%)	Solubility (%)	WVP ($g·Pa^{-1}·s^{-1}·m^{-2}·10^{-7}$)	UV barrier (%)	Transparency (%)	Young´s modulus (MPa)	Tensile strength (MPa)
PVA	1.78±0.09 a	11.8±1.07 a	357.52±25.43 b	5.1±0.55 b	0.1749±0.0109 a	17.65±1.7 b	71.87±6.92 a	1399.23±220.52 b	29.78±5.44 b
PVA-1%CNF	1.52±0.16 b	11.16±0.79 a	324.64±7.54 b	7±0.79 a	0.0697±0.0114 c	19.59±1.34 ab	71.83±7.4 a	1243.85±250.15 b	35.11±3.54 ab
PVA-3%CNF	1.26±0.07 c	10.24±1.07 a	487.47±13.38 a	7.38±0.45 a	0.0675±0.0081 c	18.91±0.06 ab	56.51±10.19 b	1492.54±197.88 ab	34.33±5.3 b
PVA-5%CNF	1.24±0.04 c	9.66±1.9 a	365.03±19.14 b	6.35±0.63 ab	0.0622±0.0062 c	19.74±1.06 ab	50.64±4.58 b	1819.97±293.51 a	41.28±1.03 a
PVA-7%CNF	1.19±0.16 c	9.82±1.03 a	357.81±45.93 b	6.18±0.84 ab	0.1322±0.0091 b	20.02±0.58 a	60.47±4 ab	1361.66±207.06 b	34.86±2.08 ab

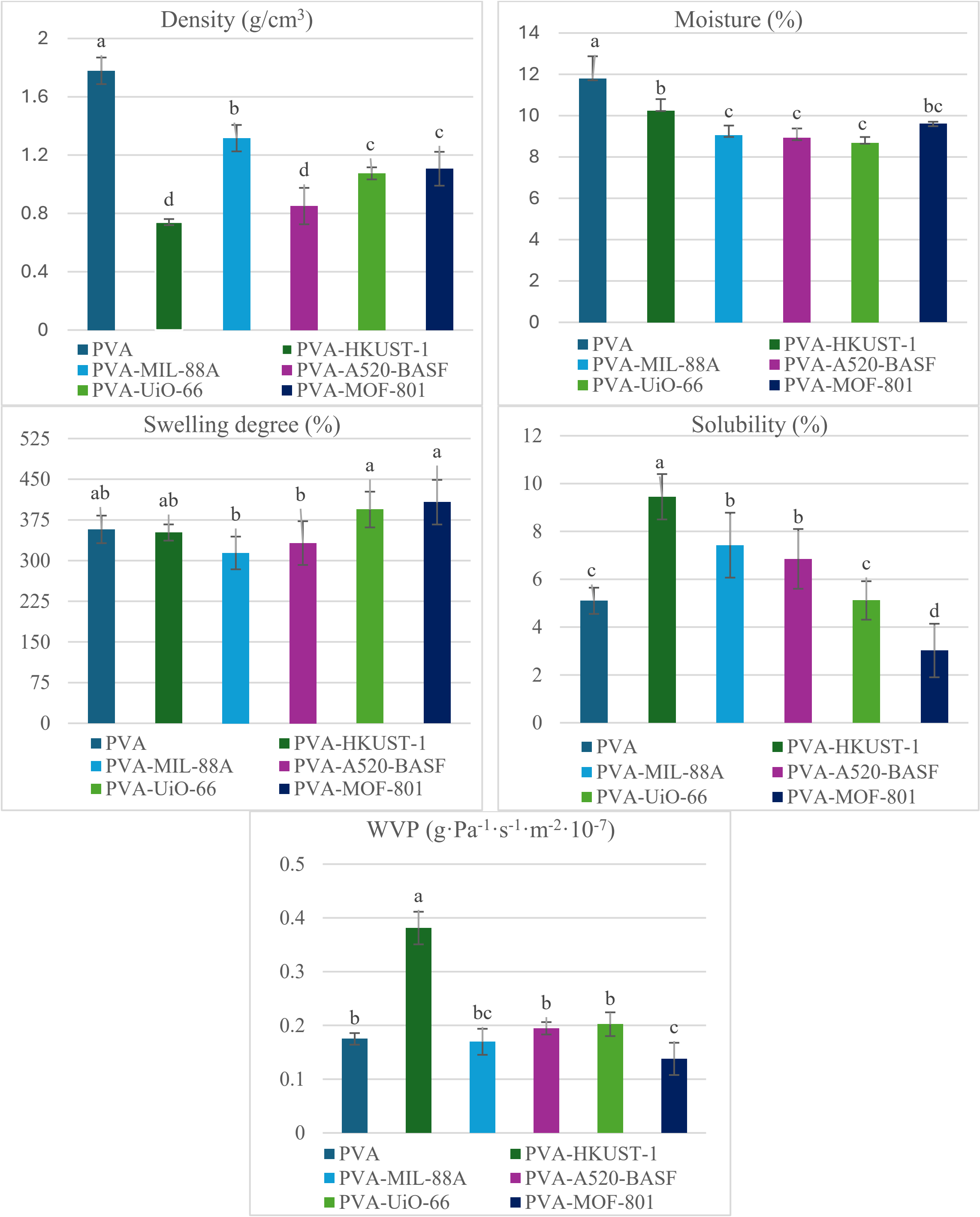

Figure S1. Physical properties (density, moisture, swelling degree, solubility and WVP) of the composite films. Different letters above bars indicate significant differences among formulations ($p \leq 0.05$).

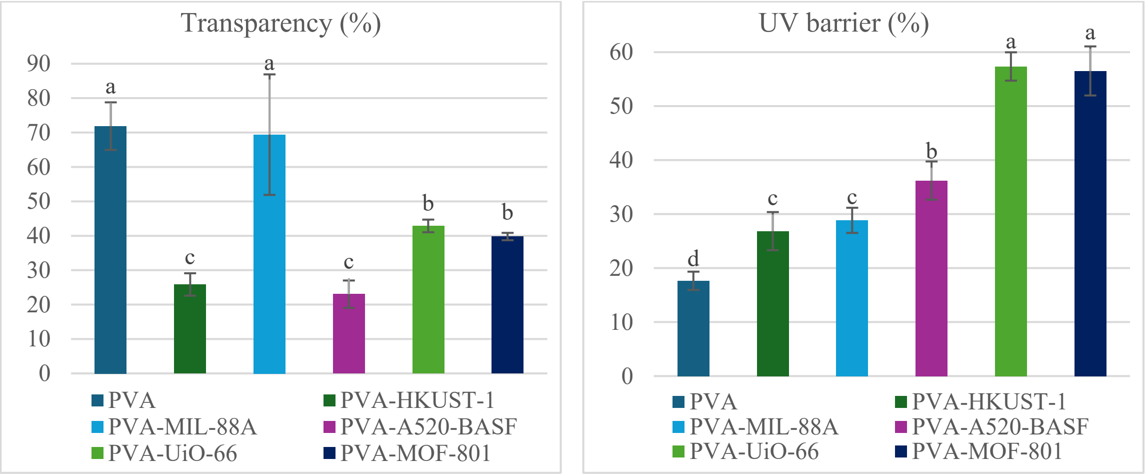

Figure S2. Transparency and UV barrier of the different composite films. Different letters above bars indicate significant differences among formulations ($p \leq 0.05$).

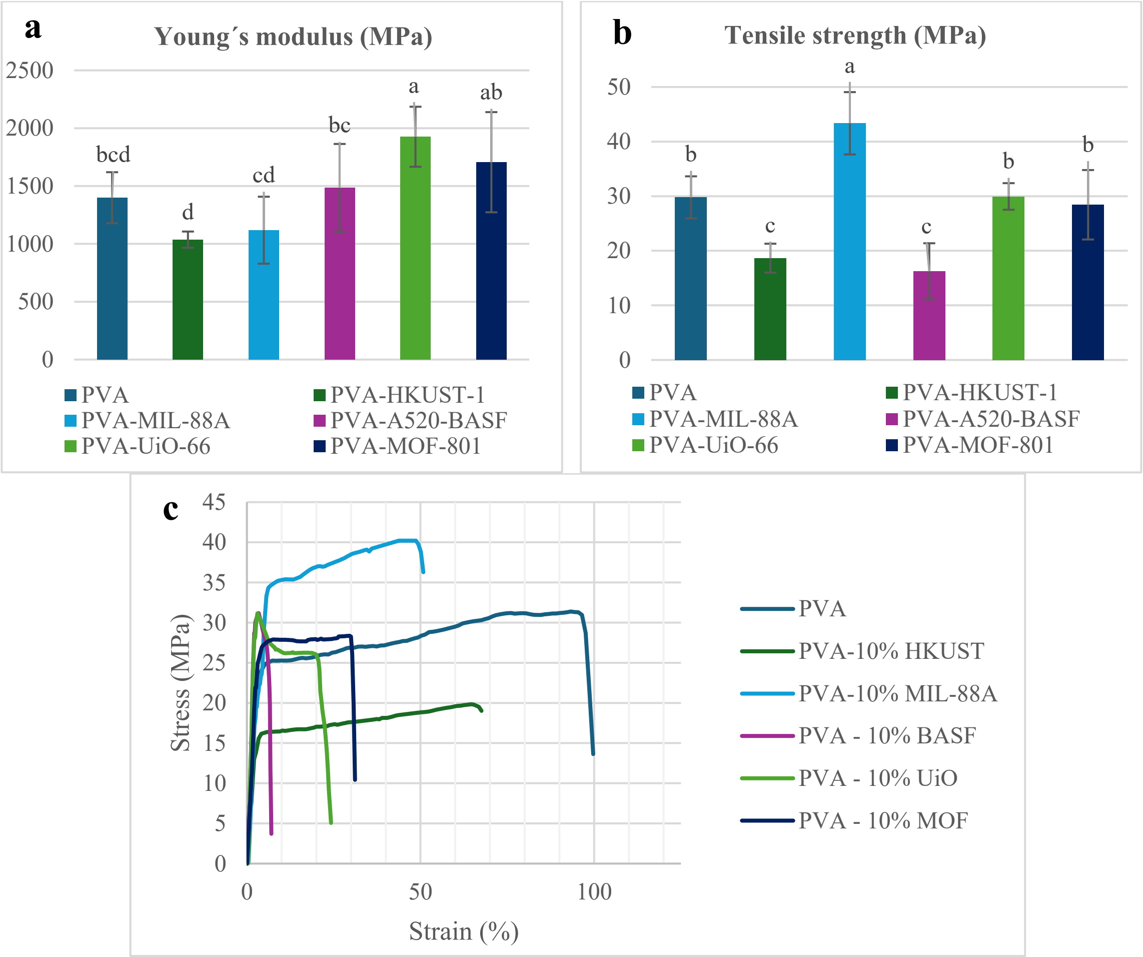

Figure S3. a) Young´s Modulus, b) tensile strength and c) stress-strain curves of PVA films with the different MOFs. Different letters above bars indicate significant differences among formulations ($p \leq 0.05$).

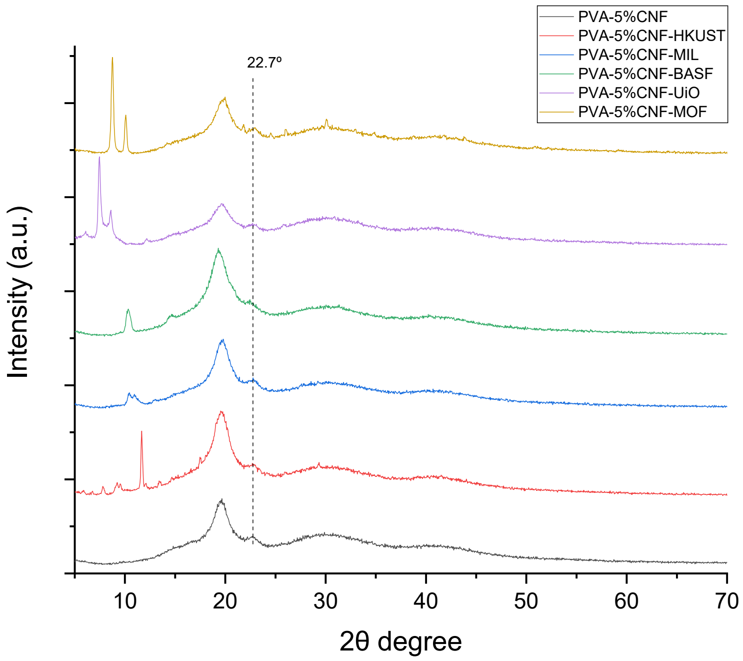

Figure S4. XRD pattern of the PVA-CNF-MOF films.

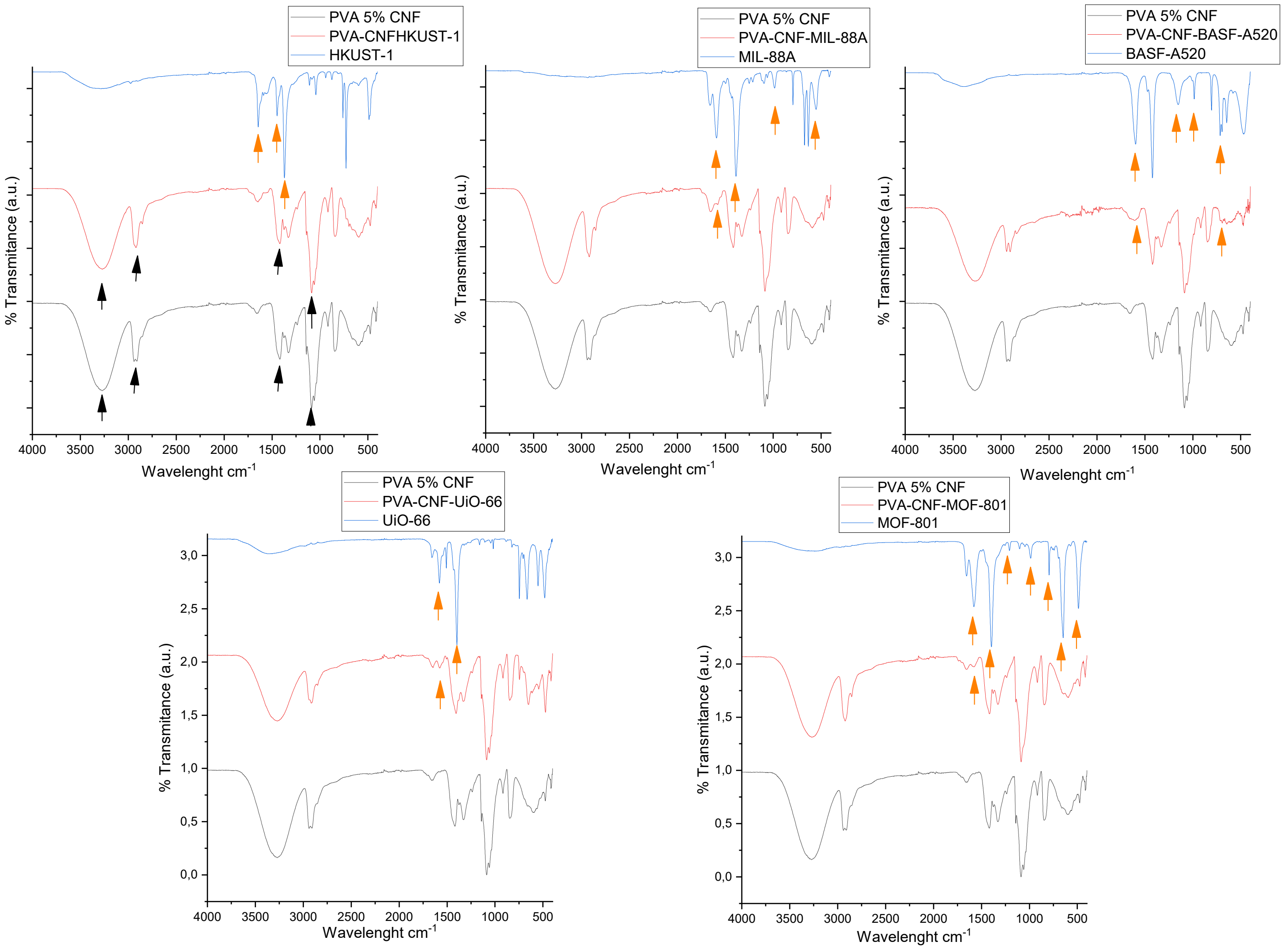

Figure S5. FTIR of the PVA-CNF-MOF composite films.
